# Aphasia Leading to the Diagnosis of Myotonic Dystrophy Type 1: A Case Report

**DOI:** 10.7759/cureus.89002

**Published:** 2025-07-29

**Authors:** Shinichiro Maeshima, Akinori Takeda, Keita Sakurai, Aiko Osawa, Hidenori Arai

**Affiliations:** 1 Education and Innovation Center, National Center for Geriatrics and Gerontology, Obu, JPN; 2 Neurology, National Center for Geriatrics and Gerontology, Obu, JPN; 3 Radiology, National Center for Geriatrics and Gerontology, Obu, JPN; 4 Rehabilitation Medicine, National Center for Geriatrics and Gerontology, Obu, JPN; 5 Internal Medicine, National Center for Geriatrics and Gerontology, Obu, JPN

**Keywords:** cognitive dysfunction, frontotemporal syndrome, myotonic dystrophy type 1, progressive aphasia, tdp-43 proteinopathy

## Abstract

Myotonic dystrophy type 1 (DM1) is a multisystem autosomal dominant disorder primarily characterized by myotonia and distal muscle weakness. Central nervous system (CNS) involvement, including cognitive, executive, and emotional dysfunctions, is increasingly being recognized; however, language impairment as an initial presentation is rare. A 50-year-old right-handed woman with a six-month history of progressive word-finding difficulty, vague speech, and social withdrawal was referred for evaluation. Neurological examination revealed distal muscle atrophy (grip strength: 5 kg right, 8 kg left) without overt dysarthria or dysphagia, and intact reflexes and coordination. Neuropsychological testing revealed fluent spontaneous speech with anomia, semantic paraphasia, impaired comprehension of longer sentences, and surface dyslexia/agraphia (Mini-Mental State Examination-Japanese: 22/30, Frontal Assessment Battery: 8/18, Raven's Colored Progressive Matrices: 28/36, Montreal Cognitive Assessment-Japanese: 21/30). Brain magnetic resonance imaging revealed left-sided frontotemporal and limbic atrophy, and ^99m^Tc-ethyl cysteinate dimer single-photon emission computed tomography showed a corresponding left-dominant hypoperfusion. Amyloid positron emission tomography (PET) scan results were negative. Two weeks later, percussion and grip myotonia emerged. Genetic analysis revealed a cytosine-thymine-guanine repeat expansion (~1500 repeats) in the myotonic protein kinase 1 gene, confirming the diagnosis of DM1. The patient's semantic‐variant primary progressive aphasia-like profile (impaired semantic processing with preserved fluency) and frontotemporal imaging findings were consistent with rare CNS phenotypes reported in DM1. Previous studies have described frontotemporal atrophy, hypoperfusion, and cognitive/emotional changes in DM1. Negative amyloid PET excluded Alzheimer-related primary progressive aphasia. The subsequent detection of myotonia and a positive family history were key to diagnosis. We conclude that this case expands the clinical spectrum of DM1 to include progressive aphasia as an initial manifestation. Clinicians should maintain a high suspicion of neuromuscular disorders and actively pursue targeted genetic testing when atypical aphasia symptoms are accompanied by distal muscle atrophy.

## Introduction

Myotonic dystrophy (MyD) is a genetic disorder characterized by generalized muscle weakness and is classified into two main types [1]. In Japan, the majority of cases are MyD type 1 (DM1), which is caused by cytosine-thymine-guanine (CTG) repeat expansion in the untranslated region of the DMPK gene [2]. This mutation leads to various clinical symptoms, including muscle stiffness (myotonia), muscle weakness, cardiac conduction abnormalities, endocrine dysfunction, and central nervous system (CNS) involvement [3]. CNS symptoms frequently include cognitive decline, executive dysfunction, daytime sleepiness, emotional dysregulation, and occasionally psychiatric manifestations [4,5].

Previous studies have highlighted that speech and language abnormalities in MyD are relatively common and multifactorial, arising from both peripheral mechanisms, such as bulbar muscle weakness and myotonia, as well as central mechanisms, including cognitive dysfunction [6]. These impairments can affect speech intelligibility, fluency, and language comprehension and expression; however, detailed neuropsychological characterization of language impairment in DM1 remains limited.

Reports focusing on progressive aphasia as the initial and isolated presentation of DM1 are extremely rare [6,7], and to our knowledge, no previous reports have described genetically confirmed DM1 initially presenting with isolated progressive aphasia. Recently, a possible association between MyD and neurodegenerative disorders has been suggested; nonetheless, our understanding of the pathophysiology and clinical spectrum of language impairment in DM1 remains incomplete.

Here, we report a rare case of DM1 that initially presented with progressive aphasia prior to the appearance of classical myotonic symptoms. We describe its clinical features, neuropsychological findings, and diagnostic challenges, and aim to underscore the importance of considering neuromuscular diseases in the differential diagnosis of progressive language impairment.

## Case presentation

A 50-year-old right-handed woman with 12 years of education who previously worked as a nurse presented with a chief complaint of progressive language impairment. Her family history included that of her father, who died at age 61 years due to MyD. Regarding her present illness, she had been working at a nursing care facility until one year ago but had quit to start her own business. She was unable to communicate effectively over the phone and frequently used vague words such as “that” and “this” because she could not recall words. She gradually avoided walking, complained of frequent fatigue, and stopped cooking. She was referred to our hospital after visiting a local psychiatric clinic.

Initial physical and neurological examination

General physical examination results were unremarkable. Neurologically, the patient was alert and well-mannered. Visual acuity, visual field, and eye movements were normal. No cranial nerve abnormalities were found, except for noticeable atrophy of the sternocleidomastoid muscle. Motor paralysis, rigidity, or tremors were not observed. However, distal muscle atrophy and generalized limb weakness were noted, with a grip strength of 5 kg in the right and 8 kg in the left upper extremities. Sensory examination results were normal, deep tendon reflexes were intact, and no pathological reflexes were elicited. Limb coordination was preserved, and no sign of dysphagia or dysarthria was found. Her gait was also normal.

Neuropsychological findings

Spontaneous speech was fluent with preserved prosody and intonation. However, naming tasks revealed anomia, primarily due to word-finding difficulty. Semantic paraphasia was noted, such as naming “*kingyo*” (goldfish) as “*koi*” (carp). Repetition of short phrases (four to five words) was preserved. When asked to describe a picture of a joyful picnic, the patient replied, “Happening? Is anyone angry in this picture?” indicating difficulty interpreting polysemous and figurative language.

**Figure 1:**
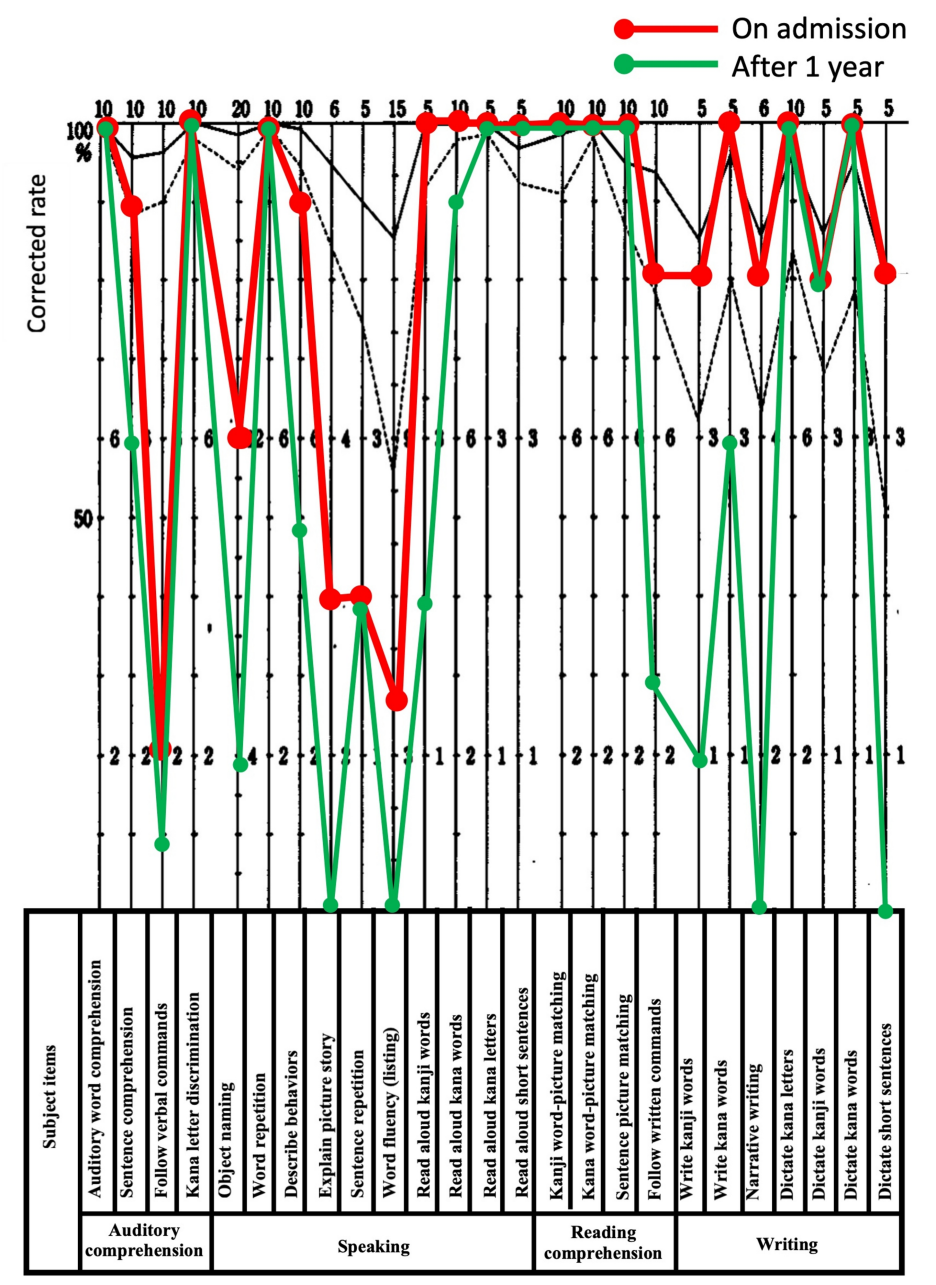
Profile of standard language test for aphasia. Summary of the patient's language profile based on the standard language test for aphasia. Naming impairment, surface dyslexia (impaired reading of *jukujikun*), and surface agraphia were evident. Comprehension was preserved for short sentences but impaired for longer and more complex constructions.

Comprehension at the word and short sentence levels was generally intact, but her understanding of longer sentences was impaired (Figure 1). Oral reading of both kanji and kana, as well as short sentences, was preserved, but reading of *jukujikun* (non-phonetic compound kanji words (see Appendix)) such as 海老 (*ebi*: shrimp), 七夕 (*tanabata*: the Star Festival), and 五月雨 (*samidare*: early summer rain) was impaired, consistent with surface dyslexia.

Writing tasks also showed preserved spontaneous writing and dictation at the word and short sentence levels. However, difficulty in writing *jukujikun *suggested surface agraphia. 

Additional cognitive assessments revealed Mini-Mental State Examination-Japanese scores of 22/30 [8], Frontal Assessment Battery 8/18 [9], Raven's Colored Progressive Matrices 28/36 [10], and Montreal Cognitive Assessment-Japanese 21/30 [11], although these scores may have been partially influenced by the aphasia.

Laboratory tests

Laboratory investigations revealed mild liver dysfunction and dyslipidemia, including elevated total and LDL cholesterol levels, as well as mildly increased transaminases and creatine kinase. Hepatitis B and C serologies were negative, and HbA1c levels were within normal limits (Table 1).

**Table 1:** Laboratory test results. ALT: alanine aminotransferase, AST: aspartate aminotransferase, γ-GTP: gamma-glutamyl transpeptidase, HB: hemoglobin, HCV: hepatitis C virus, HDL: high-density lipoprotein, LDL: low-density lipoprotein, NGSP: National Glycohemoglobin Standardization Program (HbA1c).

Parameter	Result	Reference range
Total cholesterol (mg/dL)	315	150–219
LDL cholesterol (mg/dL)	214	70-139
HDL cholesterol (mg/dL)	54	40–96
Triglycerides (mg/dL)	235	50-149
AST (U/L)	38	10–40
ALT (U/L)	42	5–40
γ-GTP (U/L)	48	<30
Creatine kinase (U/L)	271	45–163
HBs antigen	Negative	Negative
HCV antibody	Negative	Negative
HbA1c (NGSP, %)	5.4	4.6–6.2

Neuroimaging

**Figure 2:**
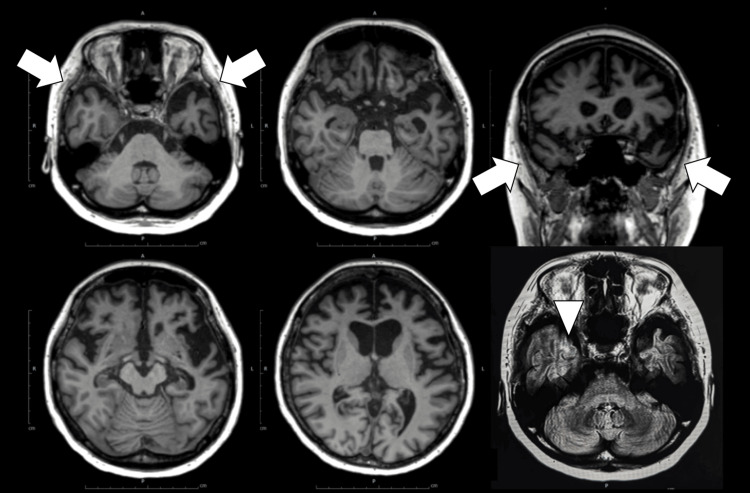
Magnetic resonance imaging findings. Head MRI showing left-dominant atrophy in the frontotemporal and limbic lobes, and insula. Characteristic knife-blade atrophy is visible, along with mild subcortical FLAIR hyperintensity in the temporal pole (arrowhead). Severe bilateral atrophy of the temporalis muscles is also noted (arrows). FLAIR: fluid-attenuated inversion recovery.

**Figure 3:**
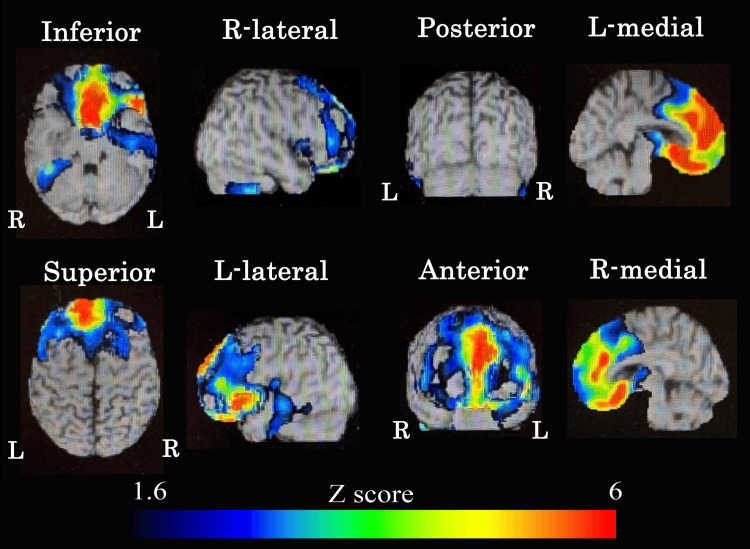
Single-photon emission computed tomography (SPECT) findings. ^99m^Tc-ECD SPECT imaging showing hypoperfusion in the left frontotemporal and limbic regions, with relative sparing of the posterior cingulate cortex and precuneus. ECD: ^99m^Tc-ethyl cysteinate dimer.

Head magnetic resonance imaging (MRI) showed left-dominant atrophy in the frontotemporal and limbic lobes and insula. A characteristic imaging finding was prominent gyral atrophy, suggestive of knife-blade atrophy. The fluid-attenuated inversion recovery (FLAIR) image showed mild hyperintensity in the subcortical white matter of the temporal pole. Additionally, severe bilateral atrophy of the temporalis muscles was also observed (Figure 2). Brain perfusion single-photon emission computed tomography (SPECT) using ^99m^Tc-ethyl cysteinate dimer (ECD) showed left-sided localized cerebral blood flow reduction in the frontal lobe, temporal lobe, and limbic system (Figure 3). The blood flow in the posterior cingulate gyrus and precuneus was preserved. Furthermore, amyloid positron emission tomography (PET) imaging was performed to investigate the possibility of Alzheimer's dementia; however, the results were negative.

Clinical course

The patient presented with progressive aphasia as her primary symptom, accompanied by distal muscle atrophy and muscle weakness, leading to the suspicion of some form of neurodegenerative disease. The patient was scheduled to visit the hospital once a week for 40 minutes of speech therapy, and her condition was monitored. After two weeks, she visited the emergency room because of lower abdominal pain and vomiting. Chest and abdominal computed tomography were performed; however, no significant findings were noted, and the patient remained under observation. At that time, impact- and grip-induced myotonia appeared, raising the suspicion of myotonic muscular dystrophy (MyD); therefore, genetic testing was conducted. The test results revealed significant elongation of the CTG repeat sequence in the non-coding region of the myotonic protein kinase gene, ranging from 1650 to 2250 bp, confirming a diagnosis of myotonic dystrophy type 1 (DM1). In general, unaffected individuals have five to 35 CTG repeats, whereas patients with DM1 typically exhibit expansions of 50 to over 1000 repeats, which correlate with disease severity and earlier onset. One year later, a language assessment using the standard language test for aphasia revealed fluent spontaneous speech and relatively preserved reading aloud ability; however, word retrieval and object naming became difficult, and auditory and reading comprehension slightly worsened. The writing of characters, both in kanji and kana, also worsened (Figure 1).

## Discussion

This case report describes an extremely rare case of progressive aphasia as the initial symptom, followed by the development of myotonia; the patient was ultimately diagnosed with myotonic dystrophy type 1 (DM1). DM1 has traditionally been recognized as a systemic disease primarily characterized by muscle symptoms; however, recent studies have highlighted the importance of CNS involvement, particularly in cognitive dysfunction, emotional disorders, and executive dysfunction [12,13]. However, DM1 presenting with language disorders, particularly progressive aphasia as the initial symptom, is extremely rare [6,7], and in cases like this, where muscle symptoms do not manifest in the early stages, diagnosis may be challenging. This case suggests that clinicians examining patients presenting with primary progressive aphasia (PPA) as the primary complaint should not overlook basic neurological findings such as muscle strength.

In this case, genetic testing confirmed the diagnosis of DM1. The test revealed a marked expansion of the CTG repeat in the DMPK gene, ranging from 1650 to 2250 base pairs. Clinically, distal muscle weakness, myotonia, and family history were important findings that supported MyD diagnosis. Language symptoms include impaired understanding of word meaning, difficulty recalling words and word substitutions, as well as fluent, syntactically intact spontaneous speech, consistent with the typical profile of the semantic-variant of primary progressive aphasia (svPPA) or semantic dementia [14,15]. The classification of these symptoms as svPPA-like was based on the international consensus criteria for primary progressive aphasia proposed by Gorno-Tempini et al. [14]. The patient exhibited the core features of svPPA, including impaired confrontation naming and single-word comprehension, in the absence of agrammatism or apraxia of speech. Surface dyslexia and dysgraphia were also observed. Although histopathological confirmation was not available, the diagnosis was supported by neuropsychological testing and imaging findings, as well as the clinical course. Additionally, imaging findings revealed atrophy from the medial temporal to the frontal lobe and localized cerebral blood flow reduction.

In DM1, various structural and functional abnormalities of the CNS have been reported, including brain atrophy centered in the frontal and temporal lobes, reduced cerebral blood flow on SPECT, and neuropsychological impairments such as executive dysfunction and emotional changes [12,16]. Cortical degeneration and cognitive dysfunction, particularly language disorders, are also known to occur [17,18], suggesting that this case may represent one such phenotype. While the incidental coexistence of svPPA cannot be theoretically ruled out, in this case, the diagnosis of DM1 was genetically confirmed. Furthermore, considering the clinical course, neuroimaging findings, and family history, it may be reasonable to interpret this as a manifestation of CNS dysfunction in DM1.

The MRI and SPECT results in this case were consistent with these reports, reaffirming that DM1 can cause selective brain damage in the frontotemporal lobe. Clinically, differentiation from frontotemporal lobar degeneration (FTLD), svPPA, and DM1-related aphasia is important when semantic aphasia is present. This patient showed severe atrophy and temporal pole subcortical hyperintensity resembling that of FTLD, particularly TAR DNA-binding protein 43 (TDP-43) proteinopathy [19]. However, in this case, the appearance of typical grip and percussion myotonia during the course of the disease, along with the presence of a family history of DM1, was clinically significant for the diagnosis. Notably, temporal muscle atrophy is often observed in patients with DM1 [3]. To date, no reports have been published on patients with genetically confirmed DM1 who also have neuropathologically verified TDP-43 proteinopathy. However, previous studies have also suggested a possible association between DM1 and tauopathy [20], which is of particular interest given the overlapping clinical features with frontotemporal dementia spectrum disorders. These findings raise the possibility that tau-related neurodegeneration may underlie certain cognitive or behavioral symptoms observed in DM1, including progressive aphasia. Additionally, negative amyloid PET findings are useful for distinguishing logopenic variant PPA (lvPPA) associated with Alzheimer's disease. Generally, lvPPA is associated with atrophy of the left temporoparietal junction and reduced cerebral blood flow, with a high prevalence of amyloid positivity. However, in this case, anterior temporal lobe-predominant atrophy and amyloid negativity supported the diagnosis of DM1 or frontotemporal dementia spectrum disorders. Although this case clinically resembled semantic-variant PPA and showed imaging findings consistent with FTLD-TDP, the genetic confirmation of DM1 and the presence of characteristic muscle symptoms suggest a DM1-related CNS manifestation. If future cases of DM1 with neuropathologically confirmed TDP-43 proteinopathy are reported, it may support the hypothesis that DM1 could partly overlap with the FTLD spectrum. At present, however, no such cases have been reported, and DM1 remains classified as a distinct multisystem disorder.

## Conclusions

This case highlights the rare presentation of DM1 with progressive aphasia as the initial symptom. Clinicians should consider neuromuscular disorders in the differential diagnosis when encountering language impairment, especially when accompanied by subtle physical signs such as muscle stiffness or weakness.

## Appendices

Note: *Jukujikun *are Japanese compound words (usually written in kanji) whose pronunciation cannot be deduced from individual readings of constituent characters. Instead, they are read as a whole, using an established, often native Japanese word. Examples include words like 海老 (*ebi*: shrimp), 七夕 (*tanabata*: the Star Festival), and 五月雨 (*samidare*: early summer rain), which are not read phonetically according to the standard *on-yomi *or *kun-yomi *of the individual kanji.

## References

[1] Thornton CA (2014). Myotonic dystrophy. Neurol Clin.

[2] Matsuura T, Minami N, Arahata H, Ohno K, Abe K, Hayashi YK, Nishino I (2012). Myotonic dystrophy type 2 is rare in the Japanese population. J Hum Genet.

[3] Wenninger S, Montagnese F, Schoser B (2018). Core clinical phenotypes in myotonic dystrophies. Front Neurol.

[4] Romeo V, Pegoraro E, Ferrati C (2010). Brain involvement in myotonic dystrophies: neuroimaging and neuropsychological comparative study in DM1 and DM2. J Neurol.

[5] Sansone V, Gandossini S, Cotelli M, Calabria M, Zanetti O, Meola G (2007). Cognitive impairment in adult myotonic dystrophies: a longitudinal study. Neurol Sci.

[6] Cardoso IL, Baptista H (2017). Myotonic dystrophy type 1 (DM1) and speech problems. JSM Commun Disord.

[7] Hanoun S, Sun Y, Ebrahimi F, Ghasemi M (2022). Speech and language abnormalities in myotonic dystrophy: an overview. J Clin Neurosci.

[8] Sugishita M, Hemmi I, Takeuchi T (2016). Reexamination of the clinical usefulness of the Japanese version of the Mini-Mental State Examination (MMSE-J). Jpn J Cogn Neurosci.

[9] Dubois B, Slachevsky A, Litvan I, Pillon B (2000). The FAB: a Frontal Assessment Battery at bedside. Neurology.

[10] Raven JC (1976). Coloured Progressive Matrices.

[11] Fujiwara Y, Suzuki H, Yasunaga M (2010). Brief screening tool for mild cognitive impairment in older Japanese: validation of the Japanese version of the Montreal Cognitive Assessment. Geriatr Gerontol Int.

[12] Okkersen K, Buskes M, Groenewoud J, Kessels RP, Knoop H, van Engelen B, Raaphorst J (2017). The cognitive profile of myotonic dystrophy type 1: a systematic review and meta-analysis. Cortex.

[13] Winblad S, Eliasdottir O, Nordström S, Lindberg C (2024). Neurocognitive disorder in Myotonic dystrophy type 1. Heliyon.

[14] Gorno-Tempini ML, Hillis AE, Weintraub S (2011). Classification of primary progressive aphasia and its variants. Neurology.

[15] Hodges JR, Patterson K (2007). Semantic dementia: a unique clinicopathological syndrome. Lancet Neurol.

[16] Huber SJ, Kissel JT, Shuttleworth EC, Chakeres DW, Clapp LE, Brogan MA (1989). Magnetic resonance imaging and clinical correlates of intellectual impairment in myotonic dystrophy. Arch Neurol.

[17] Meola G, Sansone V (2007). Cerebral involvement in myotonic dystrophies. Muscle Nerve.

[18] Modoni A, Silvestri G, Vita MG, Quaranta D, Tonali PA, Marra C (2008). Cognitive impairment in myotonic dystrophy type 1 (DM1): a longitudinal follow-up study. J Neurol.

[19] Sakurai K, Morimoto S, Oguri T (2019). Multifaceted structural magnetic resonance imaging findings in demented patients with pathologically confirmed TDP-43 proteinopathy. Neuroradiology.

[20] Hamasaki H, Maeda N, Sasagasako N (2022). Neuropathology of classic myotonic dystrophy type 1 is characterized by both early initiation of primary age-related tauopathy of the hippocampus and unique 3-repeat tauopathy of the brainstem. J Neuropathol Exp Neurol.

